# Complete Genome Sequence of the *Microbacterium* Bacteriophage Chako

**DOI:** 10.1128/mra.01251-22

**Published:** 2023-01-16

**Authors:** Mark Milhaven, Lindsey Cai, Sanjana Cherian, Kamalei Johnson, Kevin Salas Perez, Madison Blanco, Aman Garg, Jackelyn Lobatos, Corinne Mitra, Maria Strasser, Robert Harms, Susanne P. Pfeifer

**Affiliations:** a School of Life Sciences, Arizona State University, Tempe, Arizona, USA; b Center for Evolution and Medicine, Arizona State University, Tempe, Arizona, USA; c Department of Biology, St. Louis Community College, St. Louis, Missouri, USA; DOE Joint Genome Institute

## Abstract

We characterized the complete genome sequence of Chako, an obligate lytic bacteriophage with siphovirus morphology from subcluster EA1 that infects Microbacterium foliorum NRRL B-24224. Its 41.6-kb genome contains 62 putative protein-coding genes and is highly similar to that of bacteriophage HanSolo (99.26% nucleotide identity).

## ANNOUNCEMENT

Bacteriophages are a group of viruses that infect bacteria. Because they are increasingly being used to combat antibiotic-resistant bacterial infections (1, 2), characterizing bacteriophage diversity has become an important endeavor.

**FIG 1:**
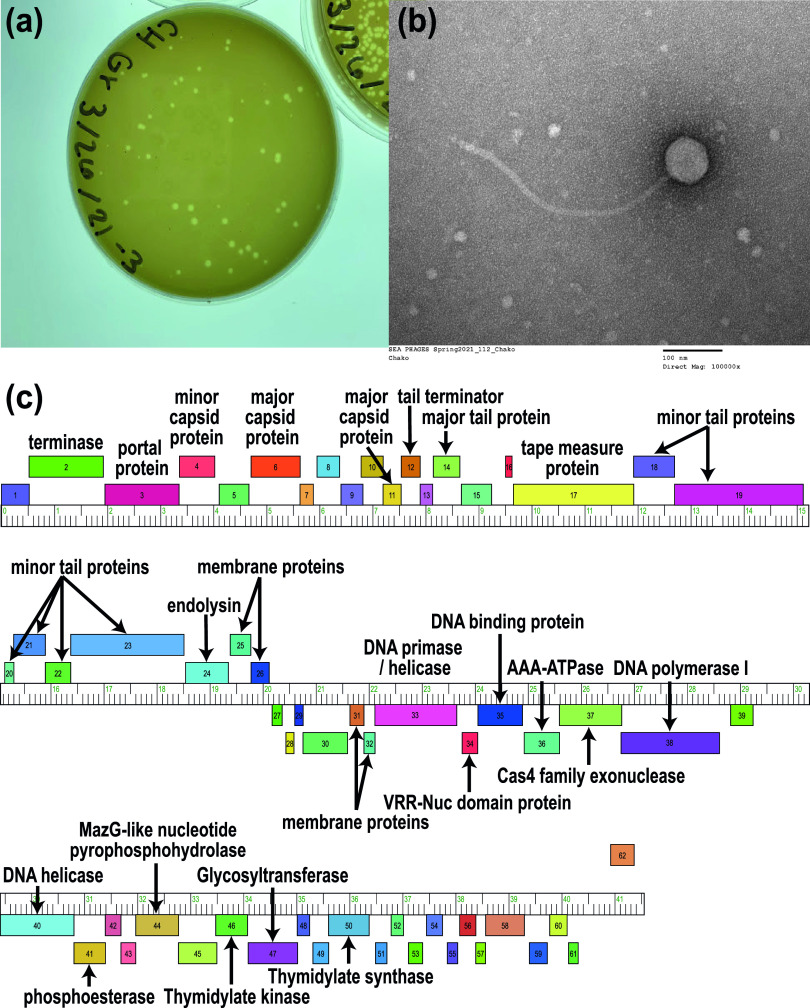
Characterization of the *Microbacterium* bacteriophage Chako. (a) Chako forms small plaques with a clear center surrounded by a turbid border. (b) An electron microscopic image highlights the *Siphoviridae* morphology of Chako, with an icosahedral capsid (diameter, ~65 nm) attached to a noncontractile tail (length, ~420 nm). The sample was viewed at an accelerating voltage of 100 kV with a 1200EX transmission electron microscope (JEOL USA, Peabody, MA) equipped with an 8-megapixel digital camera (Advanced Microscopy Techniques, Woburn, MA) after it was fixed with 1% glutaraldehyde (Ted Pella Inc., Redding, CA) on a freshly glow-discharged Formvar/carbon-coated copper grid for 10 min and stained with 1% aqueous uranyl acetate (Ted Pella Inc.) for 1 min. (c) The Chako genome shows putative protein-coding genes (boxes with numbers representing gene numbers in the genome) on the forward and reverse strands (if available, putative functional assignments are displayed above or below the ruler, respectively).

Here, we report the whole-genome sequence of Chako, a bacteriophage that was isolated from a soil sample that had been collected from a flower bed on the St. Louis Community College campus (38.56799N, 90.42246W) on 5 February 2021. The sample was taken 3 to 5 in. below the surface of the flower bed, which was covered with mulch. Following the protocols outlined in the Science Education Alliance-Phage Hunters Advancing Genomics and Evolutionary Science (SEA-PHAGES) *Phage Discovery Guide* (3), the soil sample was suspended in peptone-yeast extract-calcium (PYCa) liquid medium and shaken at 250 rpm for 1 h at 30°C, the suspension was centrifuged for 10 min at 2,000 × *g*, and the supernatant was filtered (0.22-μm pore size). A plaque assay was then performed by plating an aliquot of the filtrate on PYCa liquid medium with 250 μL of host bacteria (Microbacterium foliorum NRRL B-24224), which yielded bacteriophage Chako after 1 to 2 days at 30°C. Chako, which forms small plaques with a clear center surrounded by a turbid border (Fig. 1a), was purified through three rounds of plating. Negative-staining electron microscopy demonstrated that Chako exhibits a siphovirus morphology (Fig. 1b).

DNA was extracted from a lysate of bacteriophage Chako using a Promega Wizard DNA cleanup kit. A sequencing library was prepared using the NEBNext Ultra II library preparation kit (v3 reagents) and sequenced using the Illumina MiSeq platform, which resulted in 59,134 single-end 150-bp reads (approximately 197× coverage). Following the procedure described by Russell (4), the Chako genome was assembled using Newbler v2.9 (5), and the accuracy and completeness of the genome were checked using Consed v29.0 (6). The resulting 41,550-bp contig exhibits a GC content of 63.4%.

Following the protocols outlined in the *SEA-PHAGES Bioinformatics Guide* (7), the Chako genome was annotated using DNA Master v5.23.6 (http://cobamide2.bio.pitt.edu), GLIMMER v3.02 (8), GeneMark v2.5 (9), and Starterator v485 (https://seaphages.org/software/#Starterator), which identified 62 putative genes. Using BLASTp v2.13.0 (10) and HHpred (11) with the Protein Data Bank (PDB)/MMCIF70 (SCOPe70 v2.08 [12 August 2022]), Pfam-A v35, and NCBI Conserved Domain (CD) v3.19 databases, putative functions could be assigned to 26 genes, including several structural and assembly proteins (i.e., terminase, portal protein, minor and major capsid proteins, tail terminator, major tail protein, tape measure protein, and six minor tail proteins). Using TMHMM v2.0 (12) and SOSUI v1.11 (13), 4 additional genes could be classified as membrane proteins. ARAGORN v1.2.41 (14) and tRNAscan-SE v2.0 (15) were used to search the genome for tRNAs and transfer-messenger RNAs, but none were found. All software was run with default settings.

Based on gene content similarity of ≥35% to bacteriophages in the Actinobacteriophage Database (phagesDB), Chako was assigned to bacteriophage cluster EA1 (16, 17). Consistent with bacteriophages in this cluster, no immunity repressor or integrase functions could be identified for Chako, suggesting that it is an obligate lytic bacteriophage. Complementary multiple-sequence alignments using Kalign v3.9.01 (18) further demonstrated that Chako is closely related to cluster EA1 bacteriophages HanSolo (GenBank accession number MK967394) (99.26% identity), Clancy (GenBank accession number MK967400) (98.90% identity), and Sedgewig (GenBank accession number MT310851) (98.80% identity).

### Data availability.

Whole-genome sequencing data are available in the NCBI Sequence Read Archive (SRA) (BioProject accession number PRJNA488469 and SRA accession number SRR21924933). The annotated genome assembly is available in NCBI GenBank under accession number OP867023.
